# Brazil's landmark change on One Health, animal rights and protection

**DOI:** 10.1016/j.onehlt.2024.100847

**Published:** 2024-07-02

**Authors:** Vanessa Negrini, Paulo César Maiorka, Louise Bach Kmetiuk, Alexander Welker Biondo

**Affiliations:** a National Department of Animal Rights and Protection, Ministry of Environment and Climate Change of Brazil, Brasilia, Brazil; b Veterinary School, University of São Paulo, São Paulo, Brazil; c Department of Veterinary Medicine, Federal University of Paraná, Curitiba, Brazil

**Keywords:** Animal welfare, Environmental health, Sustainability

## Abstract

The Sustainable Development Goals (SDG) launched by the United Nations in 2015 were a global challenge calling for ending poverty, protecting the environment, and guaranteeing peace and prosperity to world citizens by 2030. Brazil has changed gears, accepted the SDG challenge and moved one step forward. On the dawn of January 1st, 2023, the very first day of President Lula's office, Brazil issued the Decree no. 11,349/2023 and created an additional SDG itself on animal health by establishing the Department of Animal Protection and Rights (DAPR). The DAPR primarily aims to establish a Federal Animal Code and other nationwide standard procedures for pet population management and mass neutering/spaying programs, services against animal cruelty, welfare meat production, meat substitutes, and enforcement of native fauna protection. Meanwhile, Brazil's new government has reduced in 48% the Amazon deforestation and mining, enforced the inspections on national parks and preserved areas, limited wild boar hunting. On top of it, Lula's G20 Bloc presidency starting this coming December has shown the Brazil commitment to reestablish its historical prominence on international conversation and diplomacy. Finally, recognition of a clear and unquestionable nexus among animal welfare, environment, and sustainability, beyond the United Nations original proposition, in a country level, particularly with still-preserved nature areas, should be understood and invested as humanity heritage.

The Sustainable Development Goals (SDG) launched by the United Nations in 2015 were a global challenge calling for poverty ending, education, environmental protection, peace, and prosperity, among the 17 main goals for world citizens by 2030 [1]. Despite being highly comprehensive, this agenda has raised three major concerns for One Health, animal rights and protection: first, a lack of an independent animal health SDG; second, animals were mentioned only once in the full document; and third, sustainability indexes have historically failed to include animal health [2].

One could argue that animal health and welfare may be indirectly accomplished in most SDGs, as improvement in human life (SDG 1, 2, 3, 8, 10, 15, and 16) and environment quality (SDG 6, 7, 11, 12, 13, 14, and 15) may positively impact the life of domestic (companion and livestock) and wildlife animals. For example, as homeless persons have shared their food with their companion animals during COVID-19 pandemics, the SDG 1 “no poverty” may provide basic infrastructure and 2 “zero hunger” enough food for vulnerable populations, which may produce animal life improvement as well. “Health and well-being” may encompass that owners rely on their pets for a better quality of life. “Quality education” may include animal welfare and responsible guardianship at schools, and “gender equality” can be interpreted as respect for all living beings. “Clear water and sanitation” should also be considered for animals, besides water-born zoonotic diseases. “Responsible consumption and production” as well as “climate actions” have demanded animal welfare and alternative protein sources. Finally, “life below water and on land” should surely include vertebrate and invertebrate animals.

Nonetheless, Brazil has changed gears, accepted the SDG challenge, and moved one step ahead on One Health, bringing animal health as part of the Government agenda. During the current 2024 massive flooding of southern Brazil, animals were included in the daily rescue lists along humans, and an emergency credit line (Federal Ordinance 1710 of May 17th, 2024) issued for municipalities to spend on animal shelters, both first ever public policy initiatives in Brazilian history. On the dawn of January 1st, 2023, the very first day of President Luiz Inácio Lula da Silva presidency (Fig.1), Brazil issued the Decree no. 11,349/2023 and created an additional SDG itself by establishing the National Department of Animal Protection and Rights (DAPR), at the Ministry of Environment and Climate Change, the first ever federal instance for animal rights. In a One Health perspective, the DAPR primarily aims to establish a Federal Animal Code and other nationwide standard regulations and procedures, through discussion and consensus with other government ministries, including pet population management and mass neutering/spaying programs, services against animal cruelty, welfare meat production, meat substitutes, and reinforcement on native fauna protection [3].

**Fig. 1:**
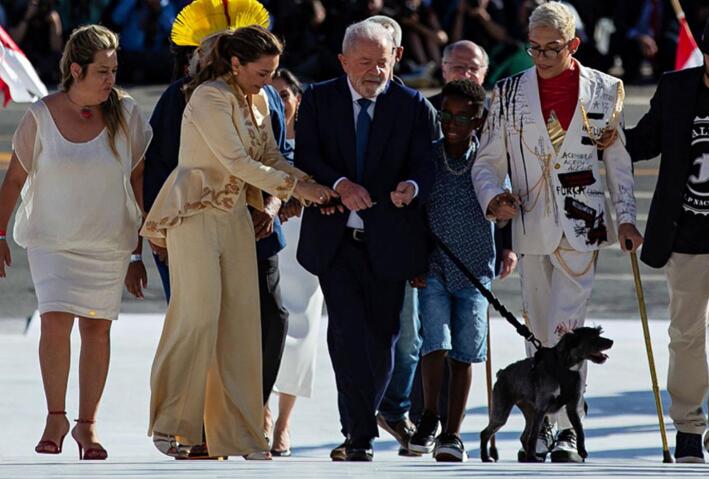
For the first time ever in Brazilian history, a dog participated in the presidential ceremony. The female, adopted named “Resistance”, walked along the president elected and his wife to take office for his third term.

Brazil has been internationally committed to the resolution launched in March 2022 by the United Nations Environment Assembly of the United Nations Environment Program (UNEP), which called for the nexus between animal welfare, the environment, and sustainable development, in addition to the quadripartite statement by FAO, WOAH, UNEP and WHO. Internally, in nine months of new government, Brazil has reduced Amazon deforestation and mining by 48%, enforced the inspections on national parks and preserved areas, limited wild boar hunting (which favored illegal native fauna hunting) [4], and included veterinarians among the indigenous health assistance professionals [5].

Brazil has been back to preservation, already shown deep commitment with environment protection and against illegal deforestation, hunting and mining. After a 14-year hiatus, the Amazon Summit 2023 brought together Brazil, Bolivia, Colombia, Ecuador, Guyana, Peru, Suriname, and Venezuela, focused on recuperating preservation and fighting criminal fire, mining, and logging; but dealing with the local structural problems and disparities and understanding regional sovereignty. In addition, invitations to Indonesia, Democratic Republic of the Congo and Republic of the Congo have elevated the discussion to a global level on addressing protection of tropical forest, biodiversity and endangered fauna and flora. On top of it, Lula's G20 Bloc presidency (started in December 2023) has shown the country's commitment to reestablish Brazil historical prominence on international dialogue and diplomacy.

The world has recently experienced fast and deep changes, particularly after the COVID-19 pandemics. One Health has become mandatory for holistic comprehension of the delicate phenomena and interrelations among human, animal, and environmental health, particularly on better understanding of environment and animal protection. One Wellbeing and One Health perceptions have grown and permeated several science fields. Finally, recognition of a clear and unquestionable nexus among animal welfare, environment, and sustainability, beyond the United Nations original proposition, within the country level, particularly with still-preserved nature areas, should be understood and invested as not only humanity but also animal and environmental legacy, as One Health heritage.

## Funding

No funding.

## CRediT authorship contribution statement

**Vanessa Negrini:** Conceptualization, Funding acquisition, Investigation, Resources, Visualization, Writing – original draft, Writing – review & editing. **Paulo César Maiorka:** Conceptualization, Visualization, Writing – original draft, Writing – review & editing. **Louise Bach Kmetiuk:** Visualization, Writing – original draft, Writing – review & editing. **Alexander Welker Biondo:** Conceptualization, Data curation, Funding acquisition, Investigation, Resources, Visualization, Writing – original draft, Writing – review & editing.

## Declaration of competing interest

None declared.

## Data Availability

No data was used for the research described in the article.
